# Stepwise Enhancement of HPV16 E6/E7 mRNA Vaccine Efficacy Using HSV-1 gD Epitope Incorporation and Immune-Modulating Agents

**DOI:** 10.21203/rs.3.rs-9890379/v1

**Published:** 2026-07-16

**Authors:** Kyle Johnson, Jane Jingting Lim, Wanfu Wu, Nguyet Tu, Shaun Xiaoliu Zhang

**Affiliations:** University of Houston; University of Houston; University of Houston; University of Houston; University of Houston

**Keywords:** HPV16 E6/E7 mRNA vaccine, HSV gD, cervical cancer, immune modulators, tumor immune microenvironment

## Abstract

Human papillomavirus (HPV)-associated malignancies remain a significant global health burden, particularly among individuals with established infection or limited access to prophylactic vaccination. Although the E6 and E7 oncogenes of high-risk HPV types represent attractive therapeutic targets, current vaccine approaches have shown limited clinical efficacy. In this study, we investigated multiple strategies to enhance the therapeutic activity of an HPV16 E6/E7 mRNA vaccine. To leverage pre-existing antiviral immune memory, we engineered an immunodominant CD4^+^ T-cell epitope derived from herpes simplex virus type 1 (HSV-1) glycoprotein D (gD) into a ubiquitin-tagged mRNA-UB-E6/E7 construct, generating an epitope-enhanced HPV16 therapeutic vaccine. The ubiquitin moiety was incorporated as an additional potentiation strategy to enhance antigen processing and presentation. Antitumor efficacy, antigen-specific T-cell responses, and tumor immune infiltration were assessed following vaccination alone or in combination with the histone deacetylase (HDAC) inhibitor entinostat or the STING agonist ADU-S100, two immunomodulatory agents known to remodel the tumor microenvironment. Incorporation of ubiquitin and HSV-1 gD CD4^+^ T-cell epitope significantly enhanced HPV-specific CD8^+^ T-cell responses and improved antitumor efficacy. Furthermore, combination therapy with either entinostat or ADU-S100 provided additional therapeutic benefit. These findings demonstrate that incorporation of a heterologous HSV-1 gD helper epitope can augment HPV E6/E7-targeted mRNA vaccination by harnessing pre-existing HSV-1-specific immune memory and enhancing CD4^+^ T-cell help. Together with ubiquitin-mediated enhancement of antigen presentation and modulation of the tumor microenvironment through HDAC inhibition or STING activation, these complementary potentiation strategies may further improve therapeutic outcomes and support the development of combinatorial immunotherapeutic approaches for HPV-associated cancers.

## Introduction

Human papillomavirus (HPV) remains one of the leading viral causes of cancer worldwide, contributing to cervical, anal, penile, vulvar, vaginal, and oropharyngeal malignancies (1). Cervical cancer is currently the fourth most common cancer among women globally (2). Persistent infection with high-risk HPV types is the central etiologic factor in these cases (3). Although prophylactic HPV vaccines have substantially improved prevention, they are unable to eliminate established infections or treat existing HPV-associated lesions and malignancies (4). Consequently, patients with persistent high-risk HPV infection, premalignant disease, or established HPV-associated malignancy still require effective therapeutic strategies including those capable of eliciting immune responses against existing disease.

Several potentiation strategies previously explored in DNA- or viral vector-based tumor vaccines have not yet been incorporated into mRNA-based HPV E6/E7 vaccines. One such strategy is the incorporation of an N-terminal ubiquitin tag to enhance E6/E7 proteasomal degradation and increase peptide availability for MHC class I loading, thereby strengthening CD8^+^ T-cell priming. (5) Previous studies demonstrated that ubiquitin fusion to HPV16 E6/E7 antigens in both DNA and viral vector-based vaccines significantly enhanced antigen-specific CD8 + T-cell responses and improved antitumor efficacy in preclinical models, (6–8) supporting the rationale for ubiquitin-mediated enhancement of cellular immunity. However, despite the rapid advancement of mRNA vaccine technology and the recent development of E6/E7-targeting mRNA vaccines, current mRNA constructs have primarily focused on codon optimization, lipid nanoparticle delivery, and incorporation of costimulatory molecules, rather than ubiquitin-directed antigen processing strategies. To date, the therapeutic potential of ubiquitin-fused HPV E6/E7 antigens has not been systematically evaluated in the context of mRNA vaccines.

In addition to efficient CD8^+^ T-cell priming, robust and durable antitumor immunity against HPV-associated malignancies also requires adequate CD4^+^ T helper cell support. CD4^+^ T cells are critical for sustaining cytotoxic T lymphocyte expansion, memory formation, and functional persistence. (9, 10) Consequently, several studies have reported that incorporation of immunodominant CD4 helper epitopes into therapeutic HPV vaccines can significantly improve vaccine immunogenicity and therapeutic efficacy. (11) Nevertheless, current approaches largely rely on de novo helper epitopes and have not explored leveraging pre-existing antiviral CD4^+^ memory repertoire established during prior common viral infections, such as herpes simplex viruses. This concept may provide a novel mechanism to rapidly recruit pre-existing helper immunity and enhance the efficacy of therapeutic HPV mRNA vaccination.

Even when robust immune responses are successfully induced following HPV E6/E7 vaccination, these responses alone may not be sufficient to achieve effective cancer therapy. One major limitation is that vaccine-induced effector T cells must efficiently traffic to and infiltrate the tumor site (12, 13). Furthermore, once these T cells enter the tumor microenvironment, they must overcome multiple immunosuppressive mechanisms that restrict their survival, proliferation, and cytotoxic function (14). Indeed, inadequate tumor infiltration by effector lymphocytes together with the highly suppressive tumor immune microenvironment represent two major obstacles to successful cancer immunotherapy (15). Tumor-associated factors such as regulatory T cells, myeloid-derived suppressor cells, inhibitory cytokines, metabolic competition, and immune checkpoint signaling collectively contribute to T-cell dysfunction and exhaustion within tumors. Consequently, substantial efforts have been devoted to developing strategies that both enhance immune cell infiltration into tumors and reverse tumor-associated immune suppression. Among these approaches, combination therapies integrating therapeutic HPV vaccines with immune checkpoint inhibitors, cytokines, epigenetic modulators, and innate immune activators have shown considerable promise (16–18).

In the present study, we developed mRNA-UB-E6/E7, an mRNA vaccine platform encoding HPV16 E6 and E7 fused to an N-terminal ubiquitin tag to enhance antigen processing and presentation. We further inserted a well-characterized CD4 helper epitope derived from the HSV-1 glycoprotein D (gD) coding sequence into mRNA-UB-E6/E7, generating the modified construct mRNA-UB-E6/E7-gD. Comparative studies demonstrated that mRNA-UB-E6/E7 elicited significantly stronger antigen-specific immune responses than the one without the ubiquitin vaccine counterpart, while incorporation of the gD CD4 helper epitope further enhanced vaccine immunogenicity and therapeutic efficacy. Moreover, combination treatment of mRNA-UB-E6/E7-gD with either the HDAC inhibitor entinostat or the STING agonist ADU-S100 further augmented antitumor activity. Collectively, these findings suggest that stepwise enhancement strategies targeting antigen presentation, CD4^+^ T-cell help, and tumor immune modulation can be effectively integrated with mRNA-UB-E6/E7-gD vaccination for the treatment of HPV-associated malignancies.

## Materials and Methods

### Mice

Five- to eight-week-old female C57BL/6 mice were obtained from Charles River Laboratories (Houston, TX) and housed under specific-pathogen-free conditions in the University of Houston animal care facility. Mice were maintained at no more than five animals per cage under a 12-h light/dark cycle, with temperature and humidity controlled at 68 to 75°F and 30 to 70%, respectively. Animals were provided with purified water and Envigo Teklad-certified rodent chow ad libitum. Following a 1-week quarantine period, mice were individually identified using numerical ear tags before experimental use. All animal procedures were performed in accordance with protocols approved by the University of Houston Institutional Animal Care and Use Committee (IACUC) and NIH guidelines for the care and use of laboratory animals.

### Cells

The TC-1 cell line, derived from C57BL/6 mouse lung epithelial cells expressing HPV16 E6 and E7, was obtained from Dr. TC Wu at Johns Hopkins University. PancO2-H7 (H7) cells were obtained from Dr. Cathy Yao at Baylor College of Medicine. TC-1 cells were maintained in RPMI 1640 medium supplemented with 10% fetal bovine serum (FBS), 1× nonessential amino acids, 2 mM L-glutamine, 1 mM sodium pyruvate, and 1% penicillin-streptomycin. To generate TC-1-GFP-Luc cells for cytotoxicity assays, parental TC-1 cells were transduced with lentiviral particles encoding a GFP-luciferase fusion reporter. Following transduction, cells were expanded, sorted, and maintained under the same culture conditions as parental TC-1 cells.

HEK293T cells were used for constructing expression and validation studies. For DNA-based validation, HEK293T cells were transfected with the indicated plasmid constructs. For mRNA-based validation, the corresponding sequences were cloned into an mRNA expression backbone, transcribed in vitro, and transfected into HEK293T cells using polyethyleneimine (PEI) at a 4:1 PEI-to-nucleic acid ratio. At 24 h post-transfection, cells were harvested in RIPA buffer, and total protein lysates were collected for downstream protein purification and quantification.

### Design and construction of vaccine plasmids

DNA sequences encoding the HPV16 E6/E7 fusion antigen were codon-optimized for murine expression and cloned into DNA and mRNA expression backbones, as previously described. For this study, the parental HPV16 E6/E7 construct was modified to include the HSV-1 glycoprotein D (gD) helper epitope gD49–82. The gD49–82 coding sequence was synthesized by GenScript and inserted within the E6 region of the HPV16 E6/E7 fusion construct using restriction enzyme-based cloning. The resulting gD epitope-containing construct was designated mRNA-E6/E7-gD.

A full-length HSV-1 gD construct was also synthesized and cloned for use in priming and validation studies. In addition, HSV-1 gH was synthesized and cloned as a control antigen. For mRNA production, inserts were cloned into a pUC-based in vitro transcription vector containing a T7 promoter, optimized untranslated regions, and a poly(A) tail. All plasmids were verified by restriction digestion and Sanger sequencing. Expression of the resulting constructs was confirmed by transfection into HEK293T cells followed by Western blot analysis.

### In vitro transcription and mRNA formulation

Messenger RNA was produced from the indicated DNA templates using the HiScribe T7 High Yield RNA Synthesis Kit (New England Biolabs) according to the manufacturer’s protocol. Transcripts were co-transcriptionally capped using CleanCap reagent and purified with the Monarch RNA Cleanup Kit (New England Biolabs) to remove residual reaction components. To reduce double-stranded RNA contaminants, purified transcripts were further processed using cellulose-based purification (20). The resulting mRNA was encapsulated in lipid nanoparticles (LNPs) via microfluidic mixing with the NanoAssemblr Spark system (Precision NanoSystems). Freshly prepared mRNA-LNP formulations were used for subsequent in vitro and in vivo experiments. LNP particle size was assessed by dynamic light scattering using a Zetasizer, with average diameters ranging from 80 to 120 nm.

### Western blot analysis of construct expression

HEK293T cells were transfected with the indicated DNA plasmids or in vitro-transcribed mRNA constructs using polyethyleneimine (PEI). At 24 h post-transfection, cells were lysed in RIPA buffer, and total protein concentrations were measured using a bicinchoninic acid (BCA) assay. Equivalent amounts of protein were separated by SDS-PAGE and transferred onto nitrocellulose membranes. HPV16 E6/E7-containing constructs were detected using an anti-HPV16 E7 antibody (NM2; Santa Cruz Biotechnology), while HSV-1 gD expression was detected using an anti-HSV-1 gD antibody (DL6; Santa Cruz Biotechnology). Membranes were then incubated with the appropriate HRP-conjugated secondary antibodies, and protein expression was visualized by chemiluminescence using an LI-COR Odyssey Fc imaging system.

### Vaccination

Mice were immunized by intramuscular injection in the hind limb using mRNA-LNP formulations. To establish HSV-1 gD-directed helper immunity, all mice first received full-length HSV-1 gD mRNA at 10 μg per dose in weeks 1 and 2. Following this priming phase, mice were assigned to treatment groups and received the indicated treatment-phase injections in weeks 3 and 4. PBS, entinostat-only, and ADU-S100-only groups received PBS during the treatment-vaccination phase, whereas vaccine groups received HSV-1 gH control mRNA, mRNA-E6/E7, mRNA-E6/E7-gD, or mRNA-UB-E6/E7-gD, depending on the indicated combination. Treatment mRNAs were administered at 10 μg per dose. Thus, all groups received the same initial HSV-1 gD priming regimen, and group differences reflect the treatment intervention administered after priming.

One week after the final treatment-phase injection, TC-1 cells were implanted subcutaneously into the right flank. Tumor growth was monitored three times per week using digital calipers, and tumor volume was calculated as (length/2) × width^2^. All mice were euthanized when tumors in the PBS control group reached the humane endpoint defined by the approved animal protocol. At the endpoint, spleens and tumors were harvested for downstream analysis. Tumors were divided for the isolation of tumor-infiltrating lymphocytes and immunohistochemical analysis.

For combination-treatment studies, the HDAC inhibitor entinostat and the STING agonist ADU-S100 were administered after tumors reached approximately 5 × 5 mm. Entinostat (MedChemExpress; cat. no. HY-12163; also known as MS-275 or SNDX-275) was administered intraperitoneally at 6 mg/kg daily. ADU-S100 [2′3′-c-di-AM(PS)2 (Rp,Rp); MedChemExpress; cat. no. HY-12885] was administered intratumorally at 10 μg per mouse for three total doses given every 3 days.

### Splenocyte preparation

Spleens and tumors were collected from euthanized mice for immune analysis. Spleens were mechanically dissociated through a 50-μm cell strainer in RPMI-based T-cell medium, and erythrocytes were lysed with ACK buffer for 1 min at room temperature. Splenocytes were then washed, resuspended in FACS buffer, filtered, counted, and used for flow cytometry or cytotoxicity assays.

Tumors were harvested at endpoint, trimmed of surrounding tissue, minced, and digested for 30 min at 37°C in RPMI containing collagenase type IV, deoxyribonuclease I, and hyaluronidase type V at final concentrations of 0.1%, 30 U/mL, and 0.01%, respectively. Digested tumors were passed through a 50-μm cell strainer and mechanically dissociated to generate single-cell suspensions. Cells were collected by centrifugation, resuspended in FACS buffer, filtered, and used for flow cytometric analysis of tumor-infiltrating lymphocytes.

For tetramer staining, an H-2D tetramer loaded with the HPV16 E7_49_–_57_peptide (RAHYNIVTF) was obtained from the NIH Tetramer Core Facility at Emory University. Splenocytes or tumor-infiltrating lymphocytes were incubated with the tetramer at a 1:100 dilution for 30 min at 4°C in the dark. Cells were then washed and stained with fluorochrome-conjugated antibodies against CD4, CD8a, CD44, and CD62L for 30 min at 4°C. The antibodies used were anti-CD4 Violet Fluor 450, clone RM4–5; anti-CD44 FITC, clone IM7; anti-CD8a PE-Cy7, clone 53 − 6.7; and anti-CD62L redFluor 710, clone MEL-14, all from Cytek. After staining, cells were washed, fixed, and analyzed using a BD FACSMelody. Data were analyzed using FlowJo.

For cytotoxicity assays, splenocytes were stimulated for 3 days in T-cell medium supplemented with recombinant murine IL-2 (200 U/mL) and anti-CD3 antibody (2 μg/mL). TC-1-GFP-Luc target cells were seeded in 96-well plates 24 h before coculture, and stimulated splenocytes were added at the indicated effector-to-target ratio. H7-GFP-Luc cells were used as antigen-negative control targets. Target cell viability was assessed by luciferase-based measurement of residual signal, and coculture supernatants were collected for IFN-γ analysis.

IFN-γ secretion was measured in CTL coculture supernatants by sandwich ELISA. Plates were coated overnight with purified anti-mouse IFN-γ capture antibody, blocked, and incubated with samples and recombinant mouse IFN-γ standards. After washing, biotinylated anti-mouse IFN-γ detection antibody and streptavidin-HRP were added. Plates were developed with TMB substrate, stopped with 1 N sulfuric acid, and absorbance was measured at 450 nm with background correction at 540 and 570 nm.

### Immunohistochemistry

Tumors were fixed in 10% neutral buffered formalin for 48 h, transferred to 70% ethanol, and subsequently paraffin-embedded. Tissue sections were prepared for immunohistochemical detection of CD4 and CD8α. Briefly, sections were deparaffinized, rehydrated, quenched for endogenous peroxidase activity, and blocked before antibody staining. Slides were incubated with primary antibodies against CD4 (EPR19514; Abcam) or CD8α (CAL38; Abcam), followed by HRP-conjugated goat anti-rabbit IgG secondary antibody. Staining was developed chromogenically, and sections were counterstained with hematoxylin.

### Statistical analysis

Data are shown as mean ± SEM. Statistical analyses were performed using GraphPad Prism 8 (GraphPad Software, San Diego, CA). Tumor growth was analyzed longitudinally using two-way ANOVA or mixed-effects analysis, as appropriate, followed by Tukey’s multiple-comparisons test. For all analyses, p < 0.05 was considered statistically significant.

## Results

### Preparation of mRNA constructs and in vitro characterization

**Figure 1:**
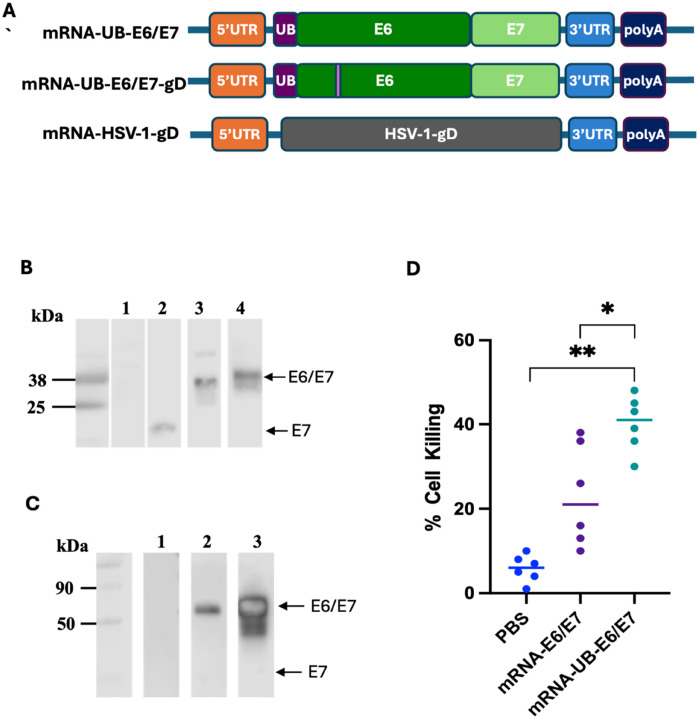
Design and characterization of HPV16 E6/E7 mRNA vaccine constructs. (A) Schematic illustration of mRNA constructs encoding HPV16 E6/E7, ubiquitin-tagged HPV16 E6/E7, ubiquitin-tagged HPV16 E6/E7 containing the HSV-1 gD CD4 epitope, and full-length HSV-1 gD. Key structural elements are indicated. (B) Western blot analysis of E6/E7 fusion protein expression in transfected HEK293 cells using an anti-HPV16 E7 antibody. Lane 1: mRNA-GFP-transfected HEK293 cells; Lane 2: TC-1 cells; Lane 3: mRNA-UB-E6/E7-transfected HEK293 cells; Lane 4: mRNA-UB-E6/E7-gD-transfected HEK293 cells. (C) Western blot analysis of HSV-1 gD expression using an anti–HSV-1 gD antibody. Lane 1: mRNA-GFP-transfected HEK293 cells; Lane 2: HSV-1-infected HEK293 cells; Lane 3: mRNA-HSV-1-gD-transfected HEK293 cells. (D) TC-1-specific CTL activity at an effector-to-target ratio of 10:1. *p < 0.05; **p < 0.01.

The design of the mRNA vaccine constructs is illustrated in Fig. 1A. In the mRNA-E6/E7 construct, the two HPV oncoprotein genes were fused in frame. In the mRNA-UB-E6/E7 construct, a ubiquitin sequence was added in frame to the N-terminus of the E6/E7 fusion gene to enhance proteasomal processing and antigen presentation. For the mRNA-UB-E6/E7-gD construct, the previously reported immunodominant CD4^+^ T-cell epitope derived from HSV-1 glycoprotein D (gD), gD_49−82_ (19), was inserted in frame within the E6 coding sequence. In addition, an mRNA construct encoding the full-length HSV-1 gD protein was generated for priming vaccination to establish gD-specific CD4^+^ memory responses prior to therapeutic vaccination. All constructs were synthesized, sequence-verified, and subsequently used for mRNA production by in vitro transcription (IVT).

To confirm expression of the transgenes encoded by the mRNA constructs, HEK293 cells were transfected with the indicated mRNAs, and protein lysates were analyzed by Western blotting using an HPV16 E7-specific monoclonal antibody (Fig. 1B). Lysates from HEK293 cells transfected with mRNA-GFP served as a negative control, whereas lysates from TC-1 tumor cells, which constitutively express HPV16 E6 and E7 proteins, served as a positive control. As expected, no E7-reactive signal was detected in lysates from the negative control cells. In contrast, a distinct E7-reactive band was observed in TC-1 lysates, confirming the specificity of the antibody. Importantly, lysates from cells transfected with either mRNA-UB-E6/E7 or mRNA-UB-E6/E7-gD exhibited abundant higher-molecular-weight E7-reactive bands, consistent with successful expression of the engineered fusion antigens. The gD expression from the mRNA-HSV-1-gD was also confirmed by western blot, and result showed that the level of gD expression from the mRNA construct is comparable to that from HSV-1 infection (Fig. 1C).

To determine whether incorporation of ubiquitin into the E6/E7 fusion construct enhances antigen presentation, C57BL/6 mice were vaccinated intramuscularly twice with either mRNA-E6/E7 or mRNA-UB-E6/E7, while mice receiving empty mRNA served as controls. Splenocytes were harvested two weeks after the second vaccination and evaluated for TC-1-specific cytotoxic activity. As shown in Fig. 1D, both mRNA vaccines induced significantly elevated antitumor immune responses compared with the control group. Notably, mice vaccinated with mRNA-UB-E6/E7 exhibited significantly higher cytotoxic T lymphocyte (CTL) activity than those receiving mRNA-E6/E7, indicating that incorporation of ubiquitin enhances antigen processing and presentation, thereby improving E6/E7-specific cellular immune responses.

### Incorporation of the HSV-1 gD CD4 epitope enhances HPV16 E6/E7-specific immune responses and antitumor efficacy in the TC-1 model

**Figure 2:**
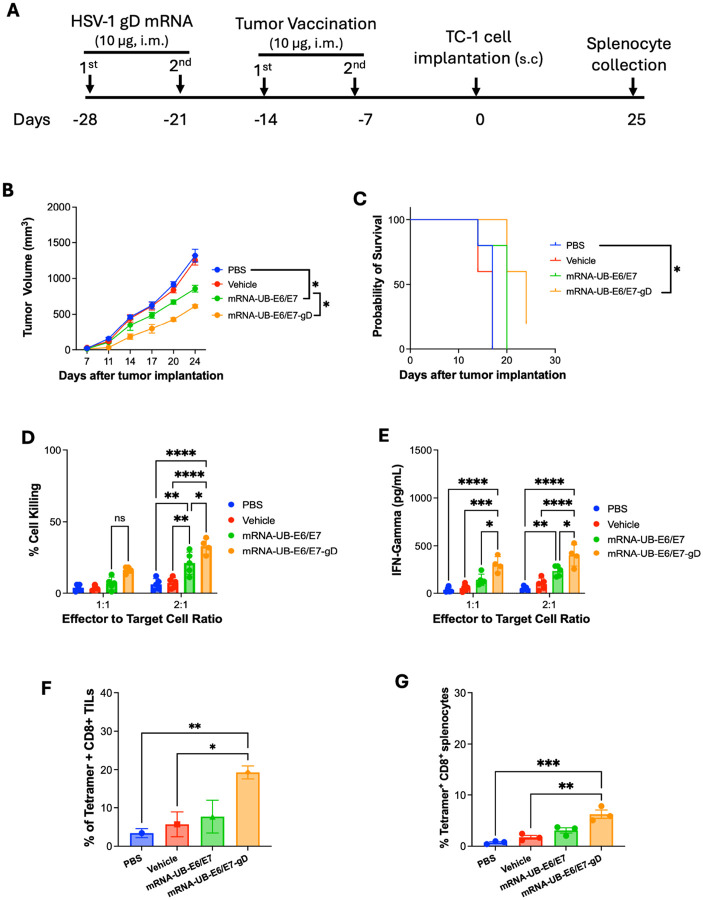
In vivo evaluation of therapeutic efficacy and immune responses following HSV-1 gD priming and HPV16 E6/E7 mRNA vaccination. (A) Experimental design. Mice were primed twice with HSV-1 gD mRNA at one-week intervals, then assigned to receive PBS, control mRNA (mRNA-HSV-1-gH), mRNA-UB-E6/E7, or mRNA-UB-E6/E7-gD. One week after the final vaccination, mice were challenged subcutaneously with TC-1 tumor cells. (B) Scatter dot plot of individual tumor volumes in each treatment group. (C) Kaplan–Meier survival analysis of treated and control mice. (D) CTL activity against TC-1 target cells measured 24 hours after assay setup at the indicated E:T ratios. (E) IFN-γ ELISA of splenocyte supernatants collected 24 hours after CTL assay setup at the indicated E:T ratios. (F,G) Flow cytometric analysis of HPV16 E7 tetramer-positive CD8+ T cells in tumor-infiltrating lymphocytes (F) and splenocytes (G). *p < 0.05; **p < 0.01; ***p < 0.001; ****p < 0.0001.

To evaluate whether incorporation of the HSV-1 glycoprotein D (gD) CD4 dominate epitope could further enhance antigen-specific immunity and therapeutic efficacy, mice were first primed with two doses of full-length HSV-1 gD mRNA before assignment into treatment groups (Fig. 2A). Mice were then vaccinated with PBS, control mRNA (mRNA-HSV-1-gH), mRNA-UB-E6/E7, or mRNA-UB-E6/E7-gD construct, followed by subcutaneous TC-1 tumor challenge. Tumor growth and survival were subsequently monitored.

As shown in Fig. 2B, both mRNA-UB-E6/E7 and mRNA-UB-E6/E7-gD produced significant antitumor effects compared with PBS and vehicle controls, demonstrating effective therapeutic immune responses. Notably, mice vaccinated with mRNA-UB-E6/E7-gD exhibited significantly improved tumor control relative to those receiving the parental mRNA-UB-E6/E7 construct. Consistently, Kaplan–Meier analysis showed prolonged survival in the mRNA-UB-E6/E7-gD group, with improved overall survival compared with both control groups and the non-gD-containing construct (Fig. 2C). Together, these findings indicate that incorporation of the HSV-1 gD immunodominant CD4 epitope enhances the therapeutic efficacy of the HPV16 E6/E7 mRNA vaccine platform.

To evaluate whether incorporation of the HSV-1 glycoprotein D (gD) CD4 epitope into mRNA-UB-E6/E7 construct could further enhance antigen-specific immune response and improve the therapeutic efficacy, mice were first primed with two doses of full-length HSV-1 gD mRNA before assignment into experimental treatment groups (Fig. 2A). This was followed by vaccination with the mRNA vaccines, in which mice were treated with PBS or vehicle control mRNA (mRNA-HSV-1-gH) as controls, or mRNA-UB-E6/E7, the helper-modified mRNA-UB-E6/E7-gD construct. Following vaccination, mice were challenged subcutaneously with TC-1 tumor cells and monitored for tumor progression and survival over time.

As shown in Fig. 2B, mice vaccinated with mRNA-UB-E6/E7 and mRNA-UB-E6/E7-gD demonstrated significant antitumor effect compared with PBS and vehicle controls, indicating efficient induction of therapeutic antitumor immunity. Moreover, mice treated with mRNA-UB-E6/E7-gD showed significantly improved tumor control compared with mice treated with the parental mRNA-UB-E6/E7 construct. This enhanced therapeutic effect was also reflected in Kaplan–Meier survival analysis, where mice receiving mRNA-UB-E6/E7 and mRNA-UB-E6/E7-gD displayed significantly prolonged survival compared with control groups, and mRNA-UB-E6/E7-gD showed the trend of improved overall survival relative to the non–gD-containing construct (Fig. 2C). Together, these findings suggest that incorporation of the HSV-1 gD CD4 dominate epitope improves the in vivo therapeutic efficacy of the HPV16 E6/E7 mRNA vaccine platform.

To determine whether improved tumor control was associated with enhanced antigen-specific immune responses, splenocytes from treated mice were evaluated for CTL activity against TC-1 target cells at low effector-to-target (E:T) ratios of 1:1 and 2:1. At an E:T ratio of 2:1, splenocytes from vaccinated mice exhibited substantially greater tumor-specific killing than those from PBS and vehicle control groups (Fig. 2D). Importantly, mice treated with mRNA-UB-E6/E7-gD showed significantly higher cytotoxic activity than those receiving the parental mRNA-UB-E6/E7 construct, indicating that inclusion of the gD helper epitope enhanced effector function. Consistent with these findings, IFN-γ ELISA of splenocyte supernatants collected 24 hours after CTL assay setup demonstrated elevated cytokine production in vaccinated groups relative to controls at both E:T ratios, with the highest IFN-γ levels observed in the mRNA-UB-E6/E7-gD group (Fig. 2E). These results indicate that incorporation of the gD helper epitope enhances systemic HPV16-specific effector immune responses.

To further assess antigen-specific CD8 + T-cell responses in both peripheral lymphoid tissues and the tumor microenvironment, HPV16 E7 tetramer staining was performed on splenocytes and tumor-infiltrating lymphocytes (TILs) (Fig. 2F,G). Flow cytometric analysis revealed increased frequencies of HPV16 E7 tetramer-positive CD8^+^ T cells in vaccinated mice compared with PBS and vehicle controls in both compartments. Moreover, mice treated with mRNA-UB-E6/E7-gD exhibited higher frequencies of tetramer-positive CD8^+^ T cells within tumors than mice receiving mRNA-UB-E6/E7, suggesting that incorporation of the gD helper epitope enhances local antigen-specific T-cell responses in the tumor microenvironment.

**Figure 3:**
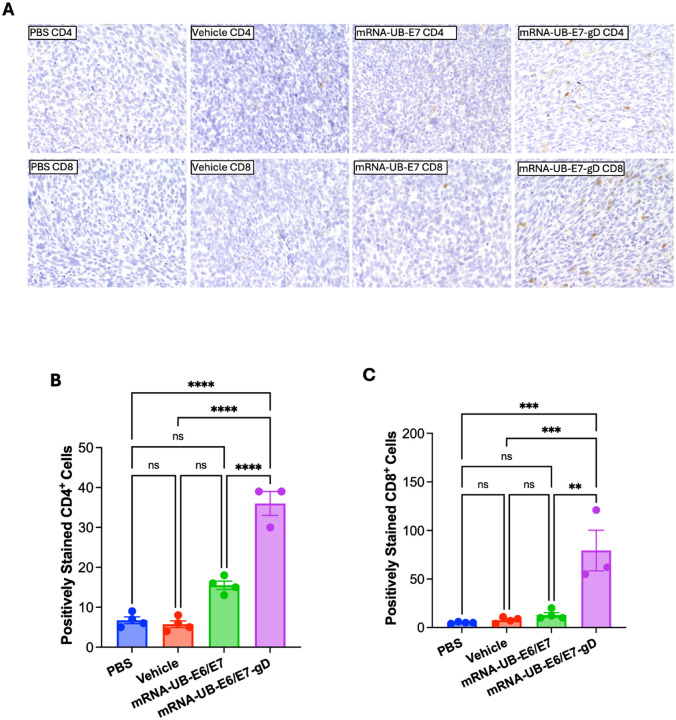
Evaluation of tumor-infiltrating T cells by immunohistochemistry (IHC). (A) Representative IHC images showing CD4^+^ and CD8^+^ T-cell infiltration in tumor sections from mice treated with PBS, vehicle control (mRNA-HSV-1-gH), mRNA-UB-E6/E7, or mRNA-UB-E6/E7-gD. Images were acquired at 20× original magnification. (B, C) Quantification of CD4^+^ (B) and CD8^+^ (C) T cells in tumor sections. Positive cells were quantified using ImageJ and expressed as the number of positive cells per microscopic field. Data were analyzed by one-way ANOVA followed by multiple-comparisons testing. Statistical significance: ns, not significant; *P < 0.05; **P < 0.01; ***P < 0.001; ****P < 0.0001.

Because effective tumor control depends not only on systemic immune activation but also on immune cell trafficking into the tumor microenvironment, tumor sections were analyzed by immunohistochemistry (IHC) for CD4^+^ and CD8^+^ T-cell infiltration (Fig. 3A). Both vaccinated groups showed increased T-cell infiltration compared with PBS and vehicle controls. Notably, tumors from mice treated with mRNA-UB-E6/E7-gD exhibited greater CD4^+^ and CD8^+^ T-cell infiltration than those from mice receiving the parental mRNA-UB-E6/E7 construct. Quantification using ImageJ confirmed significantly higher numbers of both CD4 + and CD8^+^ T cells in tumors from the mRNA-UB-E6/E7-gD group (Fig. 3B,C), while the increased T cells from mRNA-UB-E6/E7 group are predominately CD8 + cells. Together, these findings suggest that incorporation of the HSV-1 gD helper epitope enhances antitumor immunity by improving both systemic HPV16-specific immune responses and immune cell infiltration within the tumor microenvironment.

### Entinostat enhances the therapeutic efficacy of the helper-modified HPV16 E6/E7 mRNA vaccine platform.

Although incorporation of the HSV-1 gD helper epitope significantly improved the therapeutic efficacy of the mRNA-UB-E6/E7 vaccine platform, the overall antitumor effect remained incomplete. One possible explanation is the persistence of an immunosuppressive tumor microenvironment that limits the activity of vaccine-induced effector T cells. Histone deacetylase (HDAC) inhibitors such as entinostat have been shown to enhance antitumor immunity through multiple mechanisms, including increased antigen presentation, modulation of immunosuppressive cell populations, and enhancement of effector T-cell function (20, 21). We therefore evaluated whether combining entinostat with mRNA-UB-E6/E7-gD could further improve therapeutic efficacy in the TC-1 tumor model.

**Figure 4:**
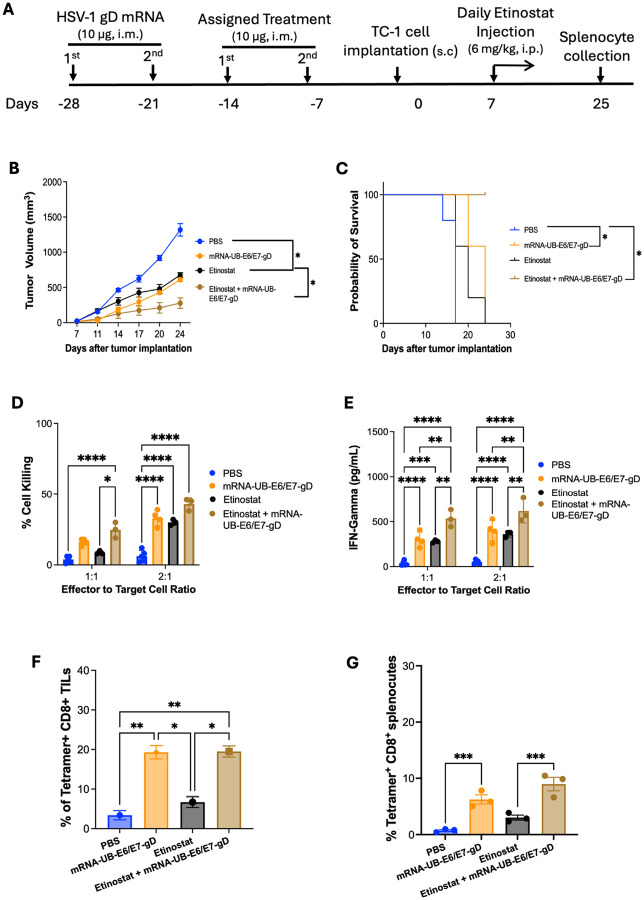
In vivo therapeutic efficacy and immune responses following Entinostat co-treatment. (A) Experimental design. All mice received two doses of HSV-1 gD mRNA at one-week intervals before assignment to treatment groups. PBS and Entinostat groups received PBS during the treatment phase, whereas vaccine groups received mRNA-UB-E6/E7-gD. One week after the final vaccination, mice were challenged subcutaneously with TC-1 cells. Entinostat (6 mg/kg) was administered daily by intraperitoneal injection beginning 7 days after tumor implantation, when tumors reached approximately 5 × 5 mm. Experimental groups included PBS, Entinostat, mRNA-UB-E6/E7-gD, and Entinostat + mRNA-UB-E6/E7-gD. (B) Scatter dot plot of individual tumor volumes. (C) Kaplan–Meier survival analysis of treated and control groups. (D) CTL activity against TC-1 target cells measured 24 hours after assay setup at the indicated effector-to-target (E) ratios. (E) IFN-γ ELISA of splenocyte supernatants collected 24 hours after CTL assay setup at the indicated E ratios. (F,G) Flow cytometric analysis of HPV16 E7 tetramer-positive CD8^+^ T cells in pooled tumor-infiltrating lymphocytes (F) and splenocytes (G). Statistical significance: ns, not significant; *p < 0.05; **p < 0.01; ***p < 0.001; ****p < 0.0001.

Again, mice were first primed with HSV-1 gD mRNA and then assigned to PBS, entinostat alone, mRNA-UB-E6/E7-gD alone, or combination treatment groups as outlined in Fig. 4A. Following TC-1 implantation, Entinostat was administered daily by intraperitoneal injection once tumors reached approximately 5 × 5 mm. Tumor growth was monitored throughout the experiment.

As shown in Fig. 4B, both Entinostat and mRNA-UB-E6/E7-gD monotherapy significantly inhibited tumor growth compared with PBS controls. Notably, Entinostat alone demonstrated therapeutic activity comparable to that of the helper-modified vaccine, consistent with previous reports of its earlier report of its antitumor activity as monotherapy (20). However, combination treatment resulted in significantly greater tumor control than either monotherapy, indicating that HDAC inhibition further potentiates the therapeutic efficacy of the vaccine platform. This enhanced effect was also reflected in Kaplan–Meier survival analysis, in which mice receiving combination therapy achieved 100% survival during the observation period, whereas all mice in the other groups died within 4 weeks (Fig. 4C).

To determine whether the improved therapeutic efficacy was associated with enhanced immune responses, TILs and splenocytes from treated mice were evaluated for TC-1-specific CTL activity. Both Entinostat monotherapy and mRNA-UB-E6/E7-gD vaccination induced measurable cytotoxicity compared with PBS controls (Fig. 4D). However, despite the markedly enhanced antitumor effect, combination therapy produced only a modest increase in CTL activity relative to either monotherapy. In contrast, IFN-γ ELISA of splenocyte supernatants collected 24 hours after CTL assay setup demonstrated substantially increased cytokine release in the combination group (Fig. 4E). Consistent with the findings in Fig. 2F,G, HPV16 E7 tetramer staining of pooled TILs and splenocytes showed significant enrichment of tetramer-positive CD8 + T cells in mice vaccinated with mRNA-UB-E6/E7-gD, whereas Entinostat alone had only marginal effects (Fig. 4F,G). However, no significant differences in tetramer-positive CD8 + T-cell frequencies were observed between the mRNA-UB-E6/E7-gD monotherapy and combination therapy groups in either tumors or spleens. Together, these findings suggest that the enhanced antitumor efficacy mediated by Entinostat is not primarily due to expansion of antigen-specific T-cell populations, but rather to improved functional activity of existing effector cells within the tumor microenvironment.

**Figure 5:**
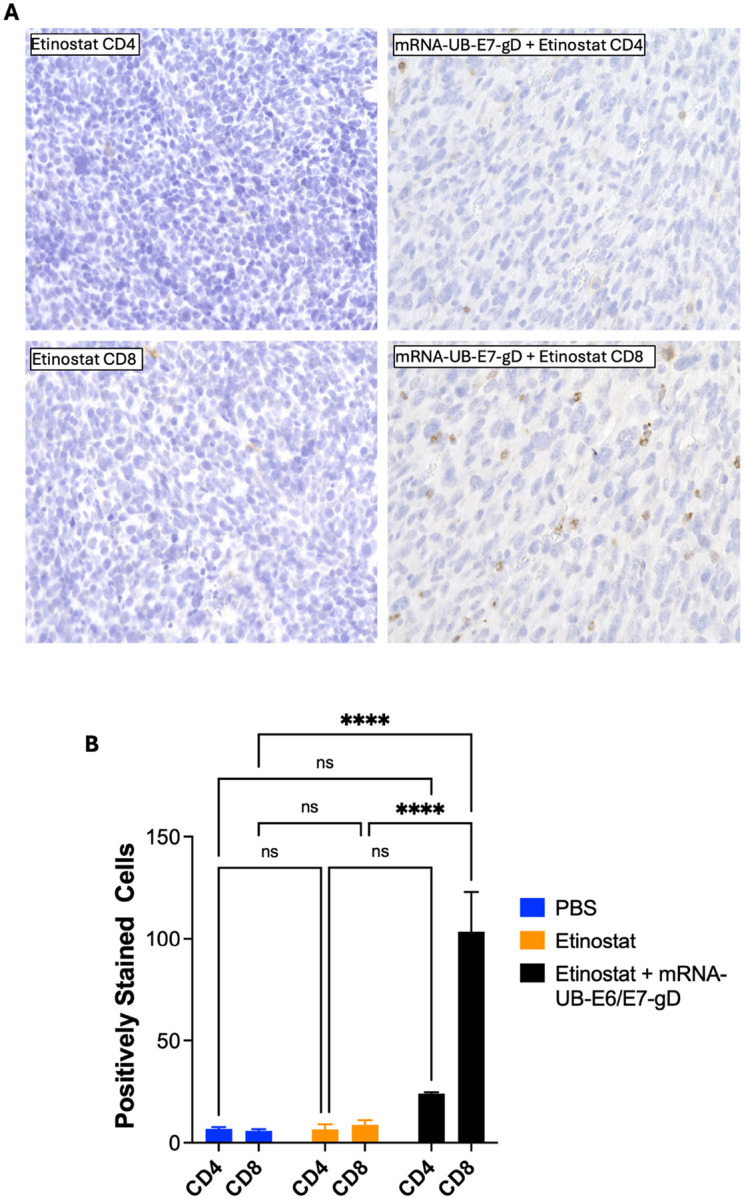
Evaluation of tumor-infiltrating T-cell responses following treatment with Entinostat co-treatment. (A) Immunohistochemistry (IHC) analysis of CD4^+^ and CD8^+^ T-cell infiltration in tumor sections (20× magnification). Representative images are shown for PBS, vehicle alone (mRNA-HSV-1-gH), mRNA-UB-E6/E7 alone, and mRNA-UB-E6/E7-gD alone treatment groups. (B) Quantification of CD4^+^ and CD8^+^positive cells from tumor sections using ImageJ software. Positive cells were counted from randomly selected microscopic fields for each tumor section, and values represent the number of positively stained cells per frame. Statistical significance is indicated as follows: Statistical significance: ns, not significant; *p < 0.05; **p < 0.01; ***p < 0.001; ****p < 0.0001.

Again, we evaluated CD4 + and CD8 + T-cell infiltration in tumor tissues by IHC (Fig. 5A,B). Similar to the results shown in Fig. 3, combination therapy induced strong CD8 + T-cell infiltration. However, a notable difference was the marked increase in CD4 + T-cell infiltration observed in tumors from mice receiving combination therapy with Entinostat. These findings suggest that the enhanced helper T-cell accumulation within the tumor microenvironment may also partly contribute to improved CD8 + T-cell functionality and the superior therapeutic efficacy of the combination treatment.

### Evaluation of STING agonist ADU-S100 in combination with mRNA-UB-E6/E7-gD

Because activation of innate immune-sensing pathways can promote inflammatory remodeling of the tumor microenvironment and enhance adaptive antitumor immunity (22), we next evaluated whether intratumoral administration of the STING agonist ADU-S100 could further improve the therapeutic efficacy of the helper-modified mRNA-UB-E6/E7-gD vaccine. STING pathway activation has been shown to increase local type I interferon signaling, promote antigen-presenting cell activation, and support downstream T-cell effector function (23). ADU-S100 is a well-characterized STING agonist capable of remodeling the tumor immune microenvironment and enhancing antitumor immune responses (23). We therefore investigated whether ADU-S100 could potentiate vaccine-induced antitumor immunity in the TC-1 tumor model.

**Figure 6:**
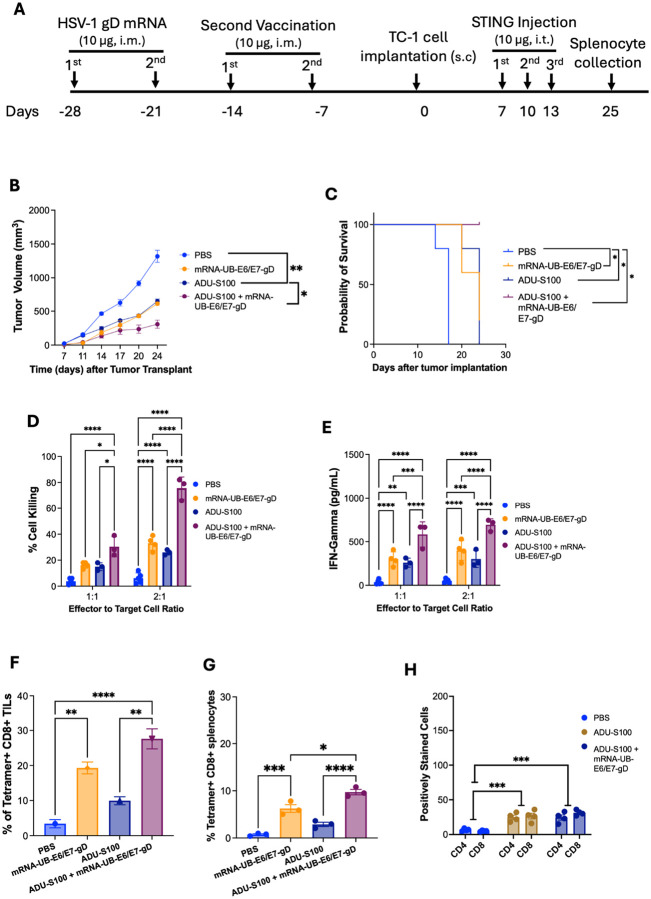
Therapeutic efficacy and immune responses following combination treatment with ADU-S100. (A) Experimental design. All mice received two doses of HSV-1 gD mRNA administered one week apart. Mice were then assigned to receive PBS, mRNA-UB-E6/E7-gD, ADU-S100, or ADU-S100 + mRNA-UB-E6/E7-gD. One week after the final vaccination, mice were challenged subcutaneously with TC-1 cells. ADU-S100 (10 μg/dose) was administered intratumorally beginning 7 days after tumor implantation, when tumors reached approximately 5 × 5 mm, for a total of three treatments. (B) Tumor volumes of individual mice at the experimental endpoint. (C) Kaplan–Meier survival analysis of the indicated treatment groups. (D) Cytotoxic T-lymphocyte (CTL) activity against TC-1 target cells measured 24 h after assay initiation. Effector-to-target (E) ratios are indicated. (E) IFN-γ production in splenocyte culture supernatants measured by ELISA 24 h after assay initiation. E ratios are indicated. (F, G) Flow cytometric analysis of HPV16 E7-specific CD8^+^ T cells in tumor-infiltrating lymphocytes (F) and splenocytes (G), determined by HPV16 E7 tetramer staining. (H) Quantification of CD4^+^ and CD8^+^ tumor-infiltrating cells by immunohistochemistry. Positive cells were quantified using ImageJ from randomly selected microscopic fields and expressed as the number of positive cells per field. Statistical significance: ns, not significant; *P < 0.05; **P < 0.01; ***P < 0.001; ****P < 0.0001.

The in vivo treatment schedule was designed similarly to the studies shown in Fig. 4 and is outlined in Fig. 5A. Antitumor efficacy and E6/E7-specific immune responses were evaluated using the same assays described for the Entinostat combination studies. As shown in Fig. 6B,C, ADU-S100 monotherapy exhibited appreciable antitumor activity, while combination treatment with mRNA-UB-E6/E7-gD further improved therapeutic efficacy. Analysis of E6/E7-specific immune responses demonstrated that combination with ADU-S100 induced stronger CTL activity than that observed with the Entinostat combination treatment (Fig. 6D,E compared with Fig. 4D,E).

A notable difference between the two combination strategies was observed in HPV16 E7 tetramer staining. Combination treatment with ADU-S100 significantly increased the frequency of tetramer-positive CD8 + T cells in both splenocytes and TILs compared with mRNA-UB-E6/E7-gD alone (Fig. 6F,G), whereas Entinostat treatment did not significantly alter tetramer-positive CD8 + T-cell frequencies (Fig. 4F,G). Another major difference was observed in tumor immune infiltration by IHC. Tumors treated with mRNA-UB-E6/E7-gD plus Entinostat were predominantly infiltrated with CD8 + T cells, whereas tumors treated with mRNA-UB-E6/E7-gD plus ADU-S100 exhibited comparatively lower CD8 + T-cell infiltration despite demonstrating strong antitumor activity.

Together, these findings indicate that although both Entinostat and ADU-S100 similarly enhanced tumor control and CTL activity against TC-1 cells, they likely exert their therapeutic effects through distinct mechanisms. Entinostat appears to primarily enhance the functional activity of existing effector T cells within the tumor microenvironment, whereas ADU-S100 more strongly promotes expansion of antigen-specific CD8 + T-cell responses through activation of innate immune signaling pathways and by improved functionality of the induced E6/E7-specific T cells and their presence in the tumor tissues.

## Discussion

Persistent infection with high-risk human papillomavirus (HPV), particularly HPV16, remains a major cause of cervical cancer and contributes substantially to other anogenital and oropharyngeal malignancies (1). Because the viral oncoproteins E6 and E7 are constitutively expressed in HPV-transformed cells and are required for maintenance of the malignant phenotype, they represent attractive tumor-specific targets for therapeutic vaccination (24). Although prophylactic HPV vaccines are highly effective in preventing infection, they do not eliminate established HPV-associated lesions or cancers. Therapeutic HPV vaccines have therefore been developed to induce E6/E7-specific cellular immunity. However, despite promising immunogenicity, therapeutic HPV vaccines have generally demonstrated limited efficacy as monotherapies in established disease, likely due to inadequate effector T-cell pool or function, insufficient tumor infiltration, and persistent immunosuppression within the tumor microenvironment (25). These limitations have driven the development of next-generation vaccine platforms designed not only to induce antigen-specific immunity but also to improve the quality and functionality of antitumor immune responses. Clinically, therapeutic HPV DNA vaccines such as VGX3100 have demonstrated efficacy in HPV-associated premalignant lesions (26), whereas treatment of advanced cancers increasingly relies on combination strategies that integrate vaccination with immune-modulating therapies. For example, the HPV16/18 E6/E7 DNA vaccine GX-188E demonstrated encouraging activity when combined with pembrolizumab in recurrent or advanced cervical cancer (27).

In the present study, we developed a multi-component mRNA-based therapeutic HPV16 vaccine platform aimed at improving antigen presentation and T-cell priming to enhance antitumor activity. First, we incorporated an N-terminal ubiquitin tag into the E6/E7 fusion construct to enhance proteasomal degradation and MHC class I antigen presentation. Although ubiquitin-mediated enhancement of antigen processing has previously been explored in DNA and viral vector vaccines (7, 28), its application to HPV16 E6/E7 mRNA vaccines has not been systematically investigated. Our results demonstrate that ubiquitin incorporation significantly enhanced CTL responses, confirming that this strategy can effectively improve antigen presentation in the context of mRNA vaccination.

We next addressed another recognized limitation of E6/E7-based vaccines: the relatively weak CD4^+^ helper T-cell responses elicited by these antigens. Several studies have shown that incorporation of exogenous helper epitopes can enhance the efficacy of HPV therapeutic vaccines (29, 30). Building on this concept, we incorporated an immunodominant HSV-1 glycoprotein D (gD) CD4 epitope into the vaccine construct. Unlike previous approaches that simply added generic helper epitopes, our strategy was designed to exploit pre-existing HSV-specific memory CD4^+^ T-cell populations, which are highly prevalent in humans. Because memory helper T cells can respond rapidly upon antigen re-encounter and augment downstream adaptive immunity, recruitment of pre-existing helper immunity represents a potentially attractive strategy for therapeutic vaccine enhancement (31).

Consistent with this rationale, incorporation of the gD helper epitope significantly improved vaccine efficacy. Compared with the parental mRNA-UB-E6/E7 construct, mRNA-UB-E6/E7-gD produced superior tumor control, prolonged survival, enhanced CTL activity, increased IFN-γ production, and higher frequencies of HPV16 E7-specific CD8^+^ T cells. Furthermore, tumors from mice receiving the gD-containing vaccine exhibited increased infiltration of both CD4^+^ and CD8^+^ T cells. Together, these findings indicate that incorporation of the HSV-derived helper epitope enhanced both systemic and intratumoral antitumor immunity. More broadly, these results provide proof-of-concept that pre-existing antiviral CD4^+^ memory responses can be leveraged to improve therapeutic cancer vaccination.

Despite these improvements, mRNA-UB-E6/E7-gD monotherapy achieved only partial tumor control. This observation is consistent with the broader experience in cancer immunotherapy, where induction of tumor-specific T cells alone is often insufficient for complete tumor eradication. Effective antitumor immunity requires not only systemic priming but also maintenance of effector function within the suppressive tumor microenvironment (32). We therefore evaluated two complementary immune-modulating strategies designed to improve the local tumor immune milieu.

The first approach involved Entinostat, a class I histone deacetylase (HDAC) inhibitor with well-documented immunomodulatory properties (33). HDAC inhibition has been associated with enhanced antigen presentation, inflammatory signaling, dendritic cell activation, and modulation of suppressive immune populations (21). In our model, Entinostat monotherapy produced measurable antitumor activity comparable to that of mRNA-UB-E6/E7-gD vaccination alone. More importantly, combination treatment significantly improved tumor control and survival relative to either monotherapy. Interestingly, this therapeutic benefit occurred without substantial increases in HPV16 E7-specific CD8^+^ T-cell frequencies or tumor T-cell infiltration. Instead, the combination primarily enhanced CTL activity and IFN-γ production, suggesting that Entinostat improved the functional quality of vaccine-induced immune responses rather than expanding antigen-specific T-cell populations. These findings are consistent with previous reports indicating that HDAC inhibitors can remodel the tumor microenvironment and enhance T-cell functionality without necessarily increasing T-cell numbers (20). Thus, Entinostat appears to function primarily as a qualitative enhancer of vaccine-induced immunity.

The second combination strategy utilized the STING agonist ADU-S100. Activation of the STING pathway promotes type I interferon production, dendritic cell activation, antigen presentation, and inflammatory remodeling of the tumor microenvironment, ultimately enhancing CD8^+^ T-cell-mediated antitumor immunity (23). Intratumoral ADU-S100 has also advanced into clinical testing, supporting its translational relevance (34). Similar to Entinostat, ADU-S100 monotherapy produced significant antitumor activity. However, combination treatment with mRNA-UB-E6/E7-gD generated the most potent therapeutic effect observed in this study, resulting in the smallest tumor burdens and complete survival throughout the observation period.

Mechanistically, ADU-S100 differed substantially from Entinostat. In addition to enhancing CTL activity and IFN-γ production, ADU-S100 significantly increased the frequency of HPV16 E7-specific CD8^+^ T cells in both splenocytes and tumor-infiltrating lymphocytes. These findings suggest that STING activation not only improves effector function but also promotes expansion and/or maintenance of antigen-specific T-cell responses. Thus, whereas Entinostat primarily enhanced the functionality of existing vaccine-induced T cells, ADU-S100 augmented both the magnitude and quality of the antitumor immune response through activation of innate immune pathways and subsequent amplification of adaptive immunity.

Collectively, the results from the Entinostat and ADU-S100 studies underscore the importance of combining therapeutic vaccination with rational immune modulation. Although both agents significantly enhanced vaccine efficacy, they appeared to do so through distinct mechanisms. Entinostat primarily improved functional antitumor activity within the tumor microenvironment, whereas ADU-S100 promoted both enhanced effector function and expansion of antigen-specific CD8^+^ T-cell responses. These findings support a model in which effective therapeutic HPV vaccination requires both robust systemic T-cell priming and favorable remodeling of the tumor microenvironment.

In conclusion, we developed a multi-component mRNA-based therapeutic HPV16 vaccine platform that combines enhanced antigen processing through ubiquitin fusion with recruitment of pre-existing helper immunity through incorporation of an HSV-1 gD CD4 epitope. This strategy significantly improved antitumor efficacy, antigen-specific CD8^+^ T-cell responses, CTL activity, IFN-γ production, and tumor immune infiltration. Furthermore, combination therapy with Entinostat or ADU-S100 produced additional therapeutic benefit through distinct but complementary immunological mechanisms. These findings provide a strong rationale for further development of therapeutic HPV vaccine strategies that integrate optimized antigen design, exploitation of pre-existing helper immunity, and targeted modulation of the tumor microenvironment.

## Data Availability

The data that support the findings of this study are contained in the supplementary files or available from the authors on request.
